# Efficacy and Safety of Acupuncture for Acute Low Back Pain in Emergency Department: A Pilot Cohort Study

**DOI:** 10.1155/2015/179731

**Published:** 2015-08-04

**Authors:** Yen-Ting Liu, Chih-Wen Chiu, Chin-Fu Chang, Tsung-Chieh Lee, Chia-Yun Chen, Shun-Chang Chang, Chia-Ying Lee, Lun-Chien Lo

**Affiliations:** ^1^Department of Chinese Medicine, Changhua Christian Hospital, Changhua 50094, Taiwan; ^2^Department of Emergency Medicine, Changhua Christian Hospital, Changhua 50094, Taiwan; ^3^Graduate Institute of Department of Bio-Industry Technology, Dayeh University, Changhua 51591, Taiwan; ^4^Graduate Institute of Statistical and Informational Science, National Changhua University of Education, Changhua 50007, Taiwan

## Abstract

*Introduction*. Low back pain (LBP) is one of the most common complaints in the emergency department (ED). There are several research articles providing evidence for acupuncture for treating chronic LBP but few about treating acute LBP. This study assessed the efficacy and safety of acupuncture for the treatment of acute LBP in the ED. *Materials and methods*. A clinical pilot cohort study was conducted. 60 participants, recruited in the ED, were divided into experimental and control groups with 1 dropout during the study. Life-threatening conditions or severe neurological defects were excluded. The experimental group (*n* = 45) received a series of fixed points of acupuncture. The control group (*n* = 14) received sham acupuncture by pasting seed-patches near acupoints. Back pain was measured using the visual analog scale (VAS) at three time points: baseline and immediately after and 3 days after intervention as the primary outcome. The secondary outcomes were heart rate variability (HRV) and adverse events. *Results*. The VAS demonstrated a significant decrease (*P* value <0.001) for the experimental group after 15 minutes of acupuncture. The variation in HRV showed no significant difference in either group. No adverse event was reported. *Conclusion*. Acupuncture might provide immediate effect in reducing the pain of acute LBP safely.

## 1. Introduction

Most adults have the experience of low back pain (LBP) in their lives [1, 2]. Low back pain is one of the most common complaints when patients visit the emergency department (ED) [3, 4]. Most cases of acute LBP are not related to any specific disease [5–7]. After checking the patients and excluding any life-threatening conditions or severe neurological deficits, sometimes the pain has still not been eased. Patients must be kept in the ED for further observation. The prolonged hospital stay due to poor pain control is a potential factor that can cause the overcrowding of the ED [8].

Pain has been regarded as the fifth vital sign (temperature, heart rate, blood pressure, and respiratory rate) recently [9], and every patient has the right to receive adequate pain management. Pain relief is an important work in the ED and there are many medications for LBP with each medication having both benefits and side effects [10–13].

Acupuncture is one of the oldest and most popular complementary alternative medicines in the world and it has been widely utilized for pain, including low back pain, osteoarthritis, headache, and cancer [14–19]. We found that there are many studies assessing the effectiveness of acupuncture for chronic LBP but few for acute LBP [15, 20].

In light of the aforementioned observation, this study focused on evaluating the efficacy and safety of acupuncture in patients with acute LBP through outpatient care in the ED.

## 2. Materials and Methods

### 2.1. Population

A clinical pilot cohort study was conducted. Patients were recruited from the emergency department (ED) of Changhua Christian Hospital (a medical center in Taiwan) with a target sample size of 60 subjects. Participants were divided into either the experimental group or control group based on their willingness to accept acupuncture treatment. All candidates received a standardized interview process. And the purpose, procedures, potential risks, and benefits of the study were explained thoroughly to the candidates. Participants had the right to withdraw from the study at any time without any consequence. All participants' written consents were obtained. The trial was conducted from March to December, 2014. The clinical trial protocol was approved by the Institute Review Board (IRB) of Changhua Christian Hospital (CCH IRB number 140214).

### 2.2. Inclusion Criteria

Participants meeting all of the following criteria will be included:

1. age 20 to 90 years, either gender;
2. visit and stay in emergency department;
3. the chief complaint being acute low back pain;
4. diagnosis with International Classification of Diseases 9th revision (ICD-9) code 724.2 Lumbago.

### 2.3. Exclusion Criteria

Participants meeting one or more of the following criteria were excluded:

1. serious comorbid conditions (e.g., life-threatening condition or severe neurological defects);
2. patients who cannot communicate reliably with the investigator or who are not likely to obey the instructions of the trial;
3. pregnancy status.

### 2.4. Baseline Assessment

#### 2.4.1. The Oswestry Disability Index (ODI)

This questionnaire (also known as Oswestry Low Back Pain Disability Questionnaire) was designed to measure a patient's functional disability resulting from spinal pain [21].

### 2.5. Interventions

Participants were divided into experimental and control groups based on their willingness to accept acupuncture treatment. The experimental group received a series of fixed points of acupuncture: Bilateral Hegu (LI4), Shousanli (LI10), Zusanli (ST36), Yanlingquan (GB34), and Taichong (LR3) [22]. Needles were correctly inserted and manually stimulated until the “De Qi” sensation was elicited. The needles stayed in place for 15 minutes. The control group received sham acupuncture by pasting seed-patches next to the same location as correct acupoints of experimental group; see Figure 2 [22].

### 2.6. Evaluations

The primary outcome evaluation was the visual analog scale (VAS) for pain. It is graded from 0 (no pain) to 10 (worst possible pain) and has proven its usefulness and clinical validity for the evaluation of pain [23]. Patients were evaluated at three timepoints in this study: before intervention (VAS-1), after intervention (VAS-2), 3 days after the intervention (VAS-3).

The secondary outcomes were heart rate variability (HRV) and adverse events. HRV was measured 2 times in this study: before the intervention and after the intervention. Many studies have shown a relation between HRV and pain [24, 25]. We tried to further identify the correlation between the intensity of pain and HRV [26, 27]. An additional secondary outcome was participants reporting any adverse events they experience, including discomfort, bruising at the sites of needle insertion, nausea, or feeling faint during or after treatment.

### 2.7. Data Analysis

First, the experimental group and control group were analyzed for comparability according to the baseline characteristics, including gender, age, body mass index (BMI), blood pressure (systole and diastole), heart rate (HR), and ODI. Chi-square test and Mann-Whitney *U* test were used to assess categorical variables. Second, in order to analyse the outcome of this study including VAS and HRV, we used Wilcoxon Signed Ranks Test and Mann-Whitney *U* test because of the sample size. All tests were conducted using SPSS (V.18.0).

## 3. Results

**Figure 1:**
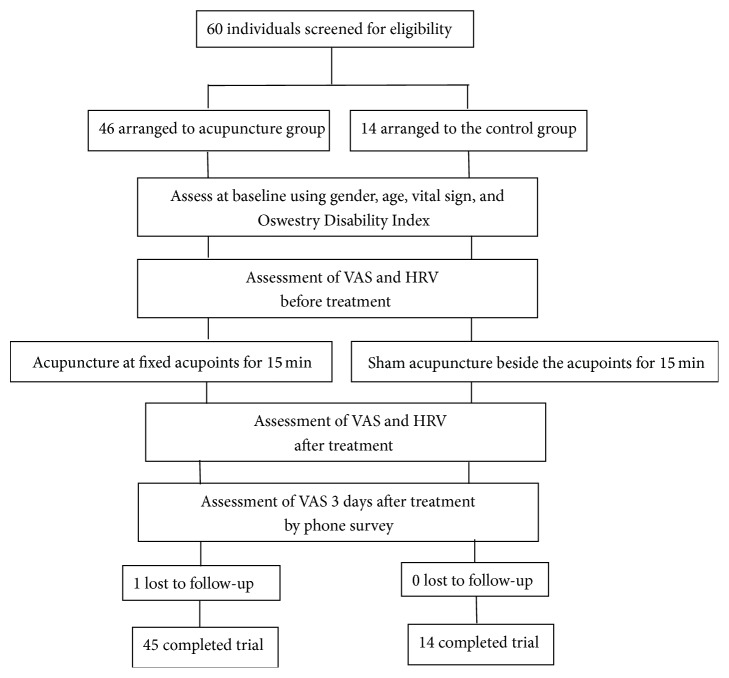
Flowchart of the study.

**Figure 2:**
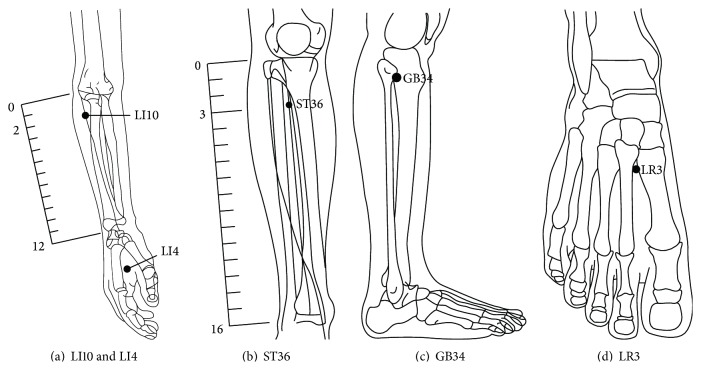
Acupoint locations (LI4, LI10, ST36, GB34, and LR3).

The flowchart of this study is presented in Figure 1.

*Participant Recruitment*. All study participants, from the emergency department (ED), were evaluated by emergency medicine specialists to exclude serious comorbid conditions and severe neurological defects, such as infection, cauda equina syndrome, and aneurysm. Sixty participants (21–89 years old, 20 men and 40 women) were recruited into the study and divided into experimental group (*n* = 46) and control group (*n* = 14). The VAS was conducted to evaluate the maintenance of the pain relieving effect by phone interview 3 days after treatment. There was 1 participant lost to follow-up in the experimental group at the 3 days after intervention timepoint.

*Baseline Characteristics*. Tables 1(a) and 1(b) show baseline participant characteristics, including gender, age, BMI, blood pressure, heart rate, and Oswestry Disability Index. The two groups were homogeneous while no significant difference was shown at baseline assessment.

*VAS*. Comparison of VAS-1 (before intervention) and VAS-2 (after intervention) indicated that there was significant difference in the experimental group (*P* < 0.001) but not in control group (*P* = 0.109). Comparison of VAS-1 and VAS-3 (3 days after intervention) found significant differences in both experimental group (*P* < 0.001) and control group (*P* = 0.011) (see Table 2).

In addition, comparison of ΔVAS1-VAS2 (changes of VAS-1 and VAS-2) between two groups also showed a significant difference (*P* < 0.001). No significant difference was observed in ΔVAS1-VAS3 (changes of VAS-1 and VAS-3) (*P* = 0.370) and ΔVAS2-VAS3 (changes of VAS-2 and VAS-3) (*P* = 0.181) (see Table 3).

Furthermore, when we do the gamma regression model with GEE method on VAS, the results also indicate a significant change after treatment in the experimental group (*P* < 0.001) but not in the control group. The VAS reduced significantly in all patients after 3 days (*P* = 0.031) (see Table 5).

*HRV*.  Table 4 shows the comparison of all parameters of HRV before and after intervention in experimental group and control group. No significant change was observed in HRV, HF%, LF%, LF/HF, VLF, LF RMSSD, and PNN50 in both groups in this study.

*Adverse Events*. No side effects were reported in this study. No patients reported bleeding, nausea, vomiting, feeling faint, or any other complication during or after intervention.

## 4. Discussion

This study was designed to demonstrate that acupuncture can benefit patients with acute LBP. Instead of recruiting participants from acupuncture outpatients, we cooperated with emergency medicine specialists in order to make the first contact with patients with acute LBP. Clinically we found that most patients with acute LBP would not be able to maintain the face-down posture during the treatment time. Therefore, all the acupoints we chose in this study were at the limbs and based on traditional Chinese medical meridian system, so patients could keep a relatively comfortable lying down position.

In the results of this study, the significant difference between VAS-1 and VAS-2 in the experimental group might prove the efficacy of acupuncture while no statistical variation was shown in control group. Another significant variation was shown in the change of VAS-1 and VAS-2 (ΔVAS1-VAS2) between two groups. It also indicated that acupuncture intervention might reduce the pain intensity. The other significant variation was between VAS-1 and VAS-3 in both groups. And it was considered as acute LBP could be mitigated through appropriate treatment without immediate recurrence [28].

HRV measures the balance of autonomic nervous system which reflects physiological, hormonal, and emotional balance within our body [29]. Many studies have proved that there are statistical differences of HRV between healthy people and patients in pain [24, 30]. But the correlation between HRV and pain intensity has not been clearly demonstrated [24, 31]. In our study, no significant difference was shown in both experimental and control group after intervention. We assume that patients might feel much less pain after 15 minutes of acupuncture (mean 6.64 ± 1.87 to 3.98 ± 1.74) but have not yet fully recovered to pain-free state.

We used the adverse event record to assess the safety. No complication was reported showing that acupuncture could be a safe treatment in patients with acute LBP. However, our study has several limitations. One limitation concerned the different number of participants between two groups. Acupuncture is a common and popular medical service in the Chinese society. Patients are usually willing to accept it. It resulted in the fact that less participants were recruited into control group when our strategy was to divide participants into two groups based on their willingness to accept acupuncture.

Another limitation was that this study was not designed as randomized blind. Considering that acupuncture is well-known in the Chinese society, it is difficult to do blinded study about acupuncture. In order to minimize the bias from this, we used seed-patches as sham acupuncture. Seed-patches are often used in auricular acupuncture. Auricular acupuncture is another well-known Chinese medical service. We tried to convince participants in control group that they were also receiving another effective treatment by pasting the seed-patches near the correct acupoints [32, 33]. Still, biases introduced by this unblinded approach cannot be ruled out.

A larger sample size in future studies is indispensable to provide well-defined types of acute low back pain for our evidence-based practice.

## 5. Conclusion

We conclude that acupuncture could provide immediate effect in reducing pain of acute low back pain significantly. The results from this study provide clinical evidence on the efficacy and safety of acupuncture to treat acute low back pain in the emergency department. Nevertheless, further larger studies are needed to replicate the findings of this study.

## Figures and Tables

**(a):** 

	Control		Acupuncture		*P* value
	*N*	%	*N*	%	
Gender	14		45		0.942
Female	7	50.0	23	51.1	
Male	7	50.0	22	48.9	

*P* value by Chi-square test.

**(b):** 

	Control (*n* = 14)			Acupuncture (*n* = 45)			*P* value
	Median	Q_1_	Q_3_	Median	Q_1_	Q_3_	
Age	65	52	79	56	46	75	0.423
BMI	26	23	28	24	22	27	0.454
SYS	134	119	137	122	117	138	0.741
DIA	76	73	78	74	71	79	0.533
HR	81	77	91	75	67	88	0.303
Oswestry							
(1) Pain intensity	3	2	4	3	3	4	0.861
(2) Personal care	2	1	5	3	2	4	0.930
(3) Lifting	5	5	5	4	3	5	0.024
(4) Walking	4	3	5	4	3	5	0.767
(5) Sitting	4	2	5	4	3	5	0.745
(6) Standing	4	3	5	4	1	4	0.099
(7) Sleeping	2	1	4	3	1	4	0.648
(8) Sex life	4	4	4	4	2	4	0.448
(9) Social life	4	3	5	4	3	5	0.205
(10) Traveling	5	3	5	5	3	5	0.853

*P* value by Mann-Whitney *U* test.

Q_1_: Percentile 25.

Q_3_: Percentile 75.

BMI, body mass index; SYS, systolic pressure; DIA, diastolic pressure; HR, heart rate.

**Table 2:** Comparison between groups of VAS before, after, and 3 days after intervention.

	Control (*n* = 14)				Acupuncture (*n* = 45)				*P* value^b^
	Median	Q_1_	Q_3_	*P* value^a^	Median	Q_1_	Q_3_	*P* value^a^	
VAS-1	5.5	4	7		7.0	5	8		0.059
VAS-2	4.5	4	6	0.109	4.0	2	5	<0.001^∗^	0.161
VAS-3	3.0	0	4	0.011^∗^	3.0	1	6	<0.001^∗^	0.465

*P* value^a^ by Wilcoxon Signed Ranks Test (take VAS1 as reference) (intergroup).

*P* value^b^ by Mann-Whitney *U* test (between groups).

Q_1_: Percentile 25.

Q_3_: Percentile 75.

VAS-1, VAS before intervention; VAS-2, VAS after intervention; VAS-3, VAS of 3 days after intervention.

^*^Statistically significant difference (*P* < 0.05).

**Table 3:** Changes in VAS between control group and acupuncture group.

	Control (*n* = 14)			Acupuncture (*n* = 45)			*P* value
	Median	Q_1_	Q_3_	Median	Q_1_	Q_3_	
ΔVAS2-VAS1	0.0	0	0	−2.0	−4	−1	<0.001^∗^
ΔVAS3-VAS1	−1.5	−3	0	−4.0	−5	−1	0.370
ΔVAS3-VAS2	−1.5	−3	0	−1.0	−3	2	0.181

*P* value by Mann-Whitney *U* test.

Q_1_: Percentile 25.

Q_3_: Percentile 75.

^*^Statistically significant difference (*P* < 0.05).

ΔVAS2-VAS1, changes of VAS-2 and VAS-1; ΔVAS3-VAS1, changes of VAS-3 and VAS-1; ΔVAS3-VAS2, changes of VAS-3 and VAS-2.

**Table 4:** Comparison of parameters of heart rate variability (HRV) before and after intervention in two groups.

Group		Before			After			*P* value
		Median	Q_1_	Q_3_	Median	Q_1_	Q_3_	
Control (*n* = 14)	HRV	39.0	33.0	49.0	31.0	26.0	45.0	0.311
	HF%	50.0	38.0	58.0	53.0	48.0	76.0	0.421
	LF%	50.0	42.0	62.0	47.0	24.0	52.0	0.421
	LF/HF	1.0	0.7	1.6	0.9	0.3	1.1	0.133
	VLF	976.0	567.0	1436.0	628.0	501.0	1098.0	0.463
	Number of irreg. hb.	8.0	0.0	48.0	2.0	0.0	13.0	0.229
	LF	305.0	92.0	509.0	154.0	54.0	199.0	0.552
	HF	258.0	196.0	323.0	224.0	133.0	428.0	0.916
	Total power	1521.0	1089.0	2401.0	961.0	676.0	2025.0	0.311
	Variance	1521.0	1089.0	2401.0	961.0	676.0	2025.0	0.311
	RMSSD	45.0	29.0	52.0	41.0	22.0	54.0	0.674
	PNN50	13.0	8.0	30.0	20.0	1.0	30.0	0.753

Acupuncture (*n* = 45)	HRV	40.0	25.0	83.0	34.0	24.0	58.0	0.273
	HF%	45.0	32.0	61.0	46.0	32.0	60.0	0.694
	LF%	55.0	39.0	68.0	53.0	39.0	68.0	0.905
	LF/HF	1.2	0.6	2.1	1.1	0.7	2.1	0.923
	VLF	891.0	382.0	4272.0	732.0	423.0	2274.0	0.561
	Number of irreg. hb.	11.0	0.0	49.0	6.5	0.0	22.0	0.158
	LF	204.0	77.0	1095.0	185.0	53.0	667.0	0.891
	HF	208.0	68.0	932.0	141.5	71.0	503.0	0.446
	Total power	1600.0	625.0	6889.0	1157.0	576.0	3364.0	0.401
	Variance	1600.0	625.0	6889.0	1157.0	576.0	3364.0	0.401
	RMSSD	34.0	22.0	75.0	32.0	22.0	59.0	0.573
	PNN50	11.0	1.0	45.0	8.5	2.0	31.0	0.353

*P* value by Wilcoxon Signed Ranks Test.

Q_1_: Percentile 25.

Q_3_: Percentile 75.

HF, high frequency; LF, low frequency; VLF, very low frequency; Number of irreg. hb., number of irregular heart beats; RMSSD, root mean square successive difference; PNN50, NN50 count divided by the total number of all NN intervals.

**Table 5:** Results of gamma regression model with GEE method on VAS.

Predictor	Coefficient	SE	Mean ratio	95% C.I.	*P* value
(Intercept)	1.701	0.368	5.478	2.665–11.259	<0.001^∗^
Age	0.001	0.002	1.001	0.997–1.005	0.637
BMI	−0.001	0.012	0.999	0.977–1.022	0.920
Gender					
Male	0.008	0.096	1.008	0.835–1.215	0.936
Female	0.000		1.000		
Group					
Acupuncture	0.156	0.098	1.169	0.964–1.417	0.113
Control	0.000		1.000		
Time					
3	−0.377	0.175	0.686	0.487–0.966	0.031^∗^
2	−0.132	0.077	0.876	0.753–1.019	0.086
1	0.000		1.000		
Interaction					
Acupuncture Time 3	0.021	0.196	1.021	0.695–1.499	0.916
Acupuncture Time 2	−0.380	0.092	0.684	0.571–0.819	<0.001^∗^
Acupuncture Time 1	0.000		1.000		
Control Time 3	0.000		1.000		
Control Time 2	0.000		1.000		
Control Time 1	0.000		1.000		

^*^Statistically significant difference (*P* < 0.05).
